# Investigation of the Mouse Infection Model for Echovirus 18

**DOI:** 10.3390/v17071011

**Published:** 2025-07-18

**Authors:** Lei Xiang, Linlin Zhai, Guanyong Ou, Wei Zhao, Yang Yang, Chenguang Shen

**Affiliations:** 1 BSL-3 Laboratory (Guangdong), Guangdong Provincial Key Laboratory of Tropical Disease Research, School of Public Health, Southern Medical University, Guangzhou 510515, China; 18701751585@163.com (L.X.); zll15649856209@163.com (L.Z.); zhaowei@smu.edu.cn (W.Z.); 2 National Clinical Research Center for Infectious Disease, Shenzhen Third People’s Hospital, Second Hospital Affiliated to Southern University of Science and Technology, Shenzhen 518112, China; guanyongou@126.com; 3 Department of Laboratory Medicine, Zhujiang Hospital, Southern Medical University, Guangzhou 510280, China; 4 Key Laboratory of Infectious Diseases Research in South China, Southern Medical University, Ministry of Education, Guangzhou 510515, China

**Keywords:** echovirus 18, mouse infection model, IFNAR1 knockout mice, pathogenesis, viral load

## Abstract

Echovirus 18, a member of the B group of enteroviruses, is a significant etiological agent of aseptic meningitis and viral encephalitis in children. In this study, we investigated the pathogenicity of E18 by establishing a mouse infection model after comparing various mouse strains and injection methods. Two-day-old IFNAR1 knockout mice infected with clinical isolates of E18 exhibited symptoms such as lethargy, hind limb paralysis, and even mortality. Similarly, some two-day-old C57BL/6J mice displayed comparable symptoms; however, the incidence was lower than that observed in IFNAR1 knockout mice. No similar symptoms were noted in any Balb/c mice. Significant pathological changes were observed in skeletal muscle, brain tissue, and other organs of symptomatic mice; among these tissues, skeletal muscle demonstrated the highest viral load. The established infection model using two-day-old IFNAR1 knockout mice provides valuable insights into further investigations regarding its pathological injury mechanisms as well as the protective effects conferred by antibodies.

## 1. Introduction

Enteroviruses (EVs) are responsible for a diverse array of human diseases, including myocarditis, pericarditis, exanthems and enanthems, conjunctivitis, and meningitis; the majority of these conditions are mild and self-limiting. Nevertheless, certain enterovirus strains possess the capacity to induce severe and potentially fatal illnesses [1]. EVs encompass enterovirus A (EV-A), B (EV-B), C (EV-C), and D (EV-D), with Echovirus type 18 (E18) serving as a prominent serotype within the EV-B category [2].

Echovirus 18 (E18) was first identified in 1955 [3], and, in recent years, there has been a multinational epidemic of this virus worldwide. Countries and regions such as the United States, Japan, India, and Germany have reported outbreaks of aseptic meningitis caused by E18, with most patients exhibiting symptoms consistent with meningitis [4,5,6,7]. In 2006, a total of 80 cases of E18 infection in young children were documented in southern Taiwan, with a case fatality rate of 4% among those diagnosed with meningitis [8]. In 2007, a case of fatal encephalitis characterized by white matter lesions in infants, attributed to Echovirus 18, was reported in France. This observation underscored the potential role of enteroviral central nervous system (CNS) infections as a contributing factor to leukoencephalitis in infants [9]. In 2015, an outbreak involving 61 pediatric cases of E18 infection presenting with aseptic encephalitis/meningitis was documented in Hebei Province, China. The primary clinical manifestations included headache, vomiting, fever, lethargy, and nausea. All patients recovered except for one individual who developed secondary epilepsy [10]. Consequently, E18 has been identified as a significant etiological agent of aseptic meningitis among young children worldwide.

The advancement of vaccines and innovative therapeutics for enterovirus (EV) infections primarily focuses on the principal pathogens associated with hand, foot, and mouth disease (HFMD), notably EV71 and CVA16. Conventional vaccines, including the inactivated EV71 vaccine, represent the most thoroughly researched and developed options for HFMD prevention. Furthermore, recombinant HFMD vaccines utilizing recombinant DNA technology have been introduced; however, these are predominantly in the early or late phases of preclinical development [11]. Animal models are essential for elucidating the mechanisms of enterovirus infections and for evaluating the efficacy of vaccines. For example, Elizabeth [12] employed an interferon-deficient murine model to investigate the effectiveness of a trivalent vaccine composed of inactivated EV-A71, CVA16, and CVA6 during both active and passive immunity assessments.

The transmission of the majority of enteroviruses is believed to occur exclusively from human to human, indicating that humans serve as the primary natural hosts for these viruses. Nevertheless, under controlled experimental conditions, numerous enteroviruses have demonstrated the ability to infect mice, cotton rats, and various non-human primates. Many of these viral infection models utilize immunocompromised mice, such as neonatal or suckling mice and IFNAR knockout mice [13,14]. For instance, the establishment of a poliovirus oral infection system utilizing human poliovirus receptors enables the generation of transgenic mice that lack alpha/beta interferon receptors [15]. Additionally, human SCARB2 transgenic mice serve as an infectious animal model for enterovirus 71 (EV71) [16].

Currently, a multitude of clinical and epidemiological studies are concentrated on E18; however, there exists a significant gap in research regarding its pathology and the mechanisms that drive E18-induced meningeal inflammation. Progress in the development of novel drugs and vaccines targeting E18 has remained stagnant, making it imperative to establish an appropriate animal model for further investigation. Previously, Li J et al. simulated Echovirus 11 (E11) infection using 2-day-old IFNAR type I knockout (IFNAR -/-) mice, which exhibit susceptibility to enteroviruses. They identified symptoms that align with those observed clinically in human cases [17]. A novel infection model employing 2-day-old IFNAR1 knockout mice has been developed to examine the pathogenicity of Echovirus 30 (E30) [18].

Building upon this foundational work, our study involved the infection of Balb/c, C57BL/6J, and IFNAR1-KO mice through various methodologies to compare clinical manifestations, organ viral loads, and histopathological alterations among the infected subjects. Ultimately, we established an E18 infection animal model utilizing 2-day-old IFNAR1-KO mice exhibiting immunocompromised functions. This research aims to elucidate the mechanisms underlying E18 infection while facilitating future vaccine development initiatives alongside novel therapeutic strategies.

## 2. Results

### 2.1. A Preliminary Analysis of the Survival Rates, Body Weights, and Clinical Scores Across Different Strains of Mice

Two-day-old Balb/c, C57, and IFNAR1-KO mice (two per group) were infected with E18 via intracranial and intraperitoneal routes, respectively, using a concentration gradient of 10^2^, 10^4^, or 10^6^ TCID_50_ (50% tissue culture infectious dose). The control group received an equivalent volume of DMEM medium. The survival rates, body weights, and clinical scores of each mouse strain were preliminarily assessed over a five-day period following infection.

**Figure 1:**
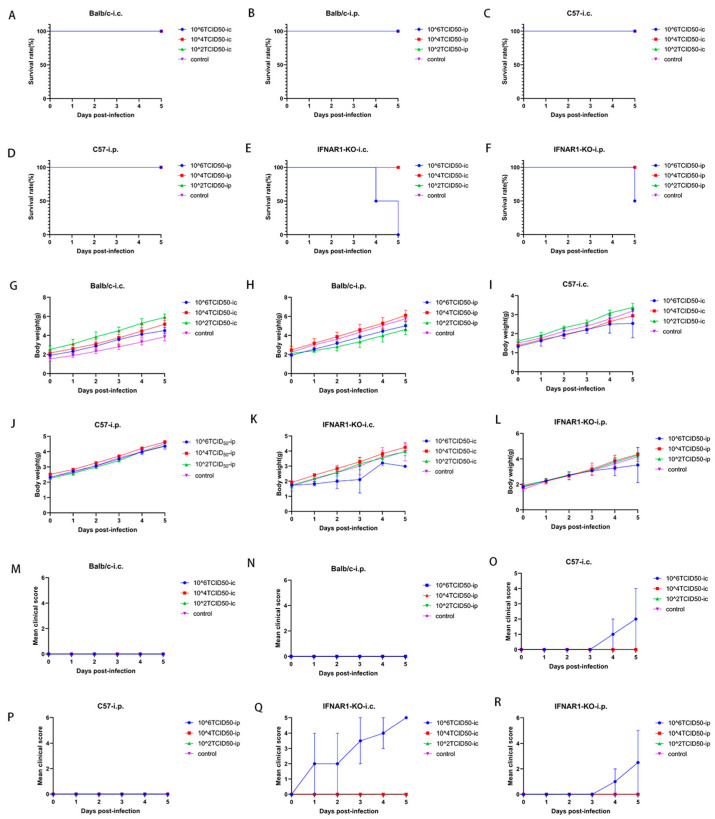
The Balb/c, C57BL/6, and IFNAR1-KO mice are infected with E18 at 2 days of age. The concentration gradients for the intracranial and intraperitoneal injections are established at 10^2^, 10^4^, and 10^6^ TCID_50_ of E18 for Balb/c, C57BL/6, and IFNAR1-KO mice, respectively (*n* = 2 per group). Control mice receive an infection-free medium as a placebo. Survival rates (**A**–**F**), body weight (**G**–**L**), and mean clinical scores (**M**–**R**) of the 2-day-old Balb/c, C57BL/6, and IFNAR1-KO mice are monitored daily following either intracranial or intraperitoneal injection.

Balb/c mice did not exhibit mortality following intracranial or intraperitoneal injection of the virus at varying concentration gradients, resulting in a 100% survival rate over five days post-infection (Figure 1A,B). Similarly, C57 mice also demonstrated no fatalities after receiving either intracranial (Figure 1C) or intraperitoneal (Figure 1D) injections. In contrast, all IFNAR1-KO mice succumbed within five days following intracranial injection, yielding a survival rate of 0% post-infection (Figure 1E). However, when subjected to intraperitoneal injection under similar conditions, these mice exhibited a survival rate of 50% over the same time frame (Figure 1F).

Balb/c mice did not demonstrate any significant body weight loss following intracranial (Figure 1G) or intraperitoneal (Figure 1H) injections of the virus at various concentration gradients. On the fifth day post-intracranial injection of 10^6^ TCID_50_ E18 in C57 mice, a slight deceleration in the upward trend of body weight was observed; however, no statistically significant differences were noted between other concentration gradients and the control group (Figure 1I). Over five days after receiving an intraperitoneal injection of 10^6^ TCID_50_ E18, no weight loss was recorded in C57 mice across all concentration gradients (Figure 1J). In contrast, IFNAR1-KO mice exhibited notable weight loss on the fifth day following intracranial injection (Figure 1K), along with a minor reduction in weight gain on the fourth day after intraperitoneal injection (Figure 1L).

There was no observed increase in the clinical disease score of Balb/c mice following intracranial or intraperitoneal injection of the virus at varying concentration gradients (Figure 1M,N). In contrast, C57 mice subjected to an intracranial injection of 10^6^ TCID_50_ E18 exhibited a notable upward trend in disease scores by the third day, with the mean disease score reaching 2 points five days post-injection (Figure 1O). Conversely, no elevation in disease scores was detected after intraperitoneal injection, indicating an absence of positive signs of illness (Figure 1P). The IFNAR1-KO mice demonstrated an increase in disease score on the first day following intracranial injection, culminating in a maximum clinical disease score of five points by day five post-injection. This suggests that all affected mice were either deceased or nearing death five days after receiving the intracranial injection (Figure 1Q). In contrast to this finding, IFNAR1-KO mice showed a rise in disease score on the third day subsequent to intraperitoneal virus administration and achieved a peak average clinical disease score of 2.5 by day five. Notably, some individuals displayed observable signs of illness during this interval (Figure 1R).

In summary, Balb/c mice did not exhibit any positive signs of disease under any experimental conditions. In C57BL/6 mice, positive signs of hind limb paralysis were observed in a subset of individuals only after the intracranial injection of 10^6^ TCID_50_ E18, which was associated with a tendency for reduced weight gain; however, no subjects succumbed to the infection. Conversely, following the intracranial administration of 10^6^ TCID_50_ E18 in IFNAR1-KO mice, all subjects displayed definitive signs of hind limb paralysis and demonstrated a significant trend toward weight loss. Notably, all individuals perished within five days post-virus injection. When IFNAR1-KO mice received an intraperitoneal injection of 10^6^ TCID_50_ E18, some individuals exhibited positive signs of hind limb paralysis; however, the trend in weight loss was less pronounced compared to those receiving intracranial injections. Additionally, some individuals did die within five days following virus administration.

### 2.2. The Survival Rates, Body Weights, and Clinical Scores of C57 Mice Subjected to Intracranial Injections, as Well as Those of IFNAR1-KO Mice Receiving Both Intracranial and Intraperitoneal Injections with 10^6^ TCID_50_ E18, Were Further Analyzed for Comparison

Given that Balb/c mice did not demonstrate any positive indicators of disease under the conditions specified in the preceding experimental session, they were excluded from consideration as experimental subjects. Following an increase in sample size (*n* = 8 for each experimental group with a 2:1 ratio of experimental to control groups), we conducted further comparisons of survival rates, clinical disease scores, and weight changes among C57 mice receiving intracranial injections of 10^6^ TCID_50_ E18 virus and IFNAR1-KO mice subjected to either intracranial or intraperitoneal injections.

**Figure 2:**
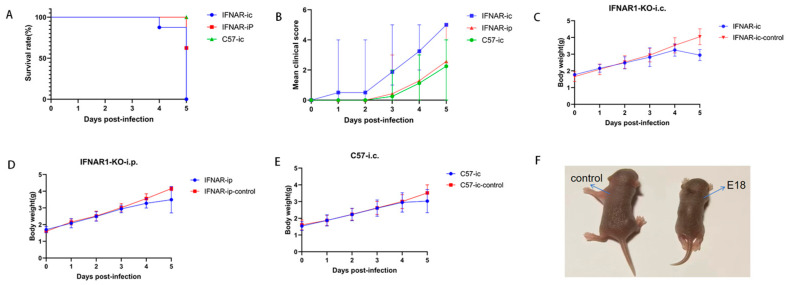
Further comparisons are performed between intracranial injections of C57 mice and IFNAR1-KO mice, as well as intraperitoneal injections of E18. Both C57 and IFNAR1-KO mice receive an injection of 10^6^ TCID_50_ E18 (*n* = 8 in each experimental group, with the ratio of experimental to control groups being 2:1). The survival rate (**A**) and clinical disease score (**B**) are recorded daily. Weight changes in IFNAR1-KO mice following intracranial injection (**C**), weight changes in IFNAR1-KO mice after intraperitoneal injection (**D**), and weight changes in C57 mice subjected to intracranial injection (**E**) are presented. Additionally, the clinical symptoms associated with E18 infection observed in 2-day-old IFNAR1-KO mice at four days post-infection (dpi) are illustrated (**F**). The grading system for clinical disease scores is defined as follows: 0—healthy; 1—lethargy or inactivity; 2—emaciation or hind limb weakness; 3—unilateral hind limb paralysis; 4—bilateral hind limb paralysis; and 5—dying or dead.

The findings regarding survival rates revealed that IFNAR1-KO mice undergoing intracranial injection exhibited the lowest survival rate after five days, with all individuals in this cohort succumbing within that period. This was succeeded by IFNAR1-KO mice receiving intraperitoneal injections, while C57 mice that were administered intracranial injections demonstrated the highest survival rate with no recorded fatalities (Figure 2A).

Clinical disease score assessments indicated that the clinical score for IFNAR1-KO mice subjected to intracranial injection was significantly elevated compared to those observed in both IFNAR1-KO mice receiving intraperitoneal injection and C57 mice undergoing intracranial injection (Figure 2B). Weight change analysis revealed that, relative to the control group, weight gain across all three administration methods was reduced; however, only the IFNAR1-KO intracranial injection at a dose of 10^6^ TCID_50_ E18 resulted in significant weight loss by day five post-injection (Figure 2C–E). A comparison of hind limb paralysis symptoms between IFNAR1-KO mice and control groups is depicted in Figure 2F.

### 2.3. A Comparative Analysis of Viral Load Between C57 and IFNAR1-KO Mice at 5 Days

#### Post-Infection (dpi) Following Intracranial and Intraperitoneal Administration of 10^6^ TCID_50_ E18

Following the administration of 10^6^ TCID_50_ E18 at 5 days post-infection (dpi), we evaluated the viral load in tissue homogenate supernatants from two mice per group, specifically C57 and IFNAR1-KO strains. Notably, intracranial injection methods for both IFNAR1-KO and C57 mice resulted in clinical disease scores of ≥4, indicative of hind limb paralysis symptoms. Conversely, no clinical signs were observed in individuals receiving intraperitoneal injections among the C57 cohort. The quantification of virus copy numbers in tissue homogenates was performed using qPCR, facilitating pairwise comparisons of viral loads between different injection techniques within the same mouse strain as well as across distinct mouse strains under identical injection conditions (bilateral *t*-test).

**Figure 3:**
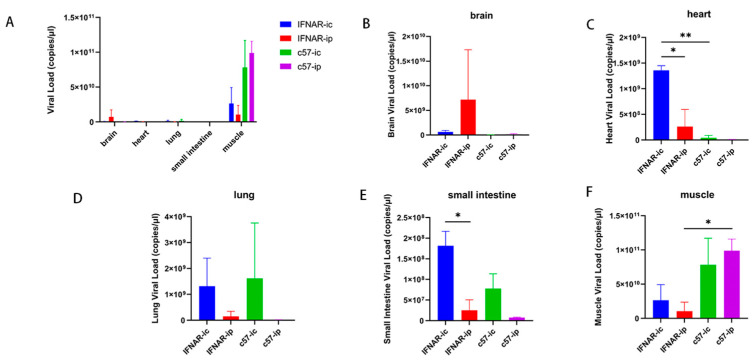
Following the reverse transcription of RNA extracted from tissue homogenates, RT-qPCR is utilized to quantify the viral load at each tissue site in C57 and IFNAR1-KO mice after the intracranial and abdominal injection of 10^6^ TCID_50_ at 5 days post-infection (dpi) (**A**). The viral loads in brain tissue (**B**), heart tissue (**C**), lung tissue (**D**), small intestine (**E**), and muscle tissue (**F**) were compared. A two-tailed *t*-test is employed to evaluate the significance of the data; * indicates *p* < 0.05, ** indicates *p* < 0.01. The figure illustrates qPCR results obtained from three biological replicates, each involving two mice per group conducted simultaneously. Data are presented as mean values ± standard error of the mean derived from two mice with three biological replicates.

The test results revealed that the viral load in the hind limb muscle was significantly elevated compared to that observed in other tissues across all injection protocols (Figure 3A). No statistically significant differences were found in viral load within brain tissue among all groups of mice (Figure 3B). In assessing the viral load in the heart, the intracranial injection method for IFNAR1-KO mice exhibited a markedly higher level than that seen with intraperitoneal injection. Additionally, this intracranial approach for IFNAR1-KO mice showed a substantial increase when compared to C57BL/6J mice (Figure 3C). There were no significant differences between mouse groups regarding viral load detection in lung tissue (Figure 3D). When evaluating viral load in the small intestine, intracranial injection of IFNAR1-KO demonstrated significantly higher levels than intraperitoneal injection; however, no significant differences were noted among other groups (Figure 3E). In terms of muscle tissue viral load detection, the intraperitoneal injection mode for C57BL/6J was significantly greater than that of IFNAR1-KO mice while showing no significant difference among other groups (Figure 3F). In the control group without E18 injection, Ct values for virus detection at all sites exceeded 35, and no virus was detected.

### 2.4. Histopathological Examination of IFNAR1 Knockout Mice Exhibiting Bilateral Hind Limb Paralysis Following E18 Viral Infection

E18, with a viral titer of 10^6^ TCID_50_/mL, was administered to IFNAR1-KO mice via intracranial injection of 50 μL. Upon reaching a clinical score of 4 (indicative of positive signs of bilateral hind limb paralysis), the animals were euthanized. Subsequently, the tissues were surgically excised, processed for histological analysis, and stained with hematoxylin and eosin (HE).

**Figure 4:**
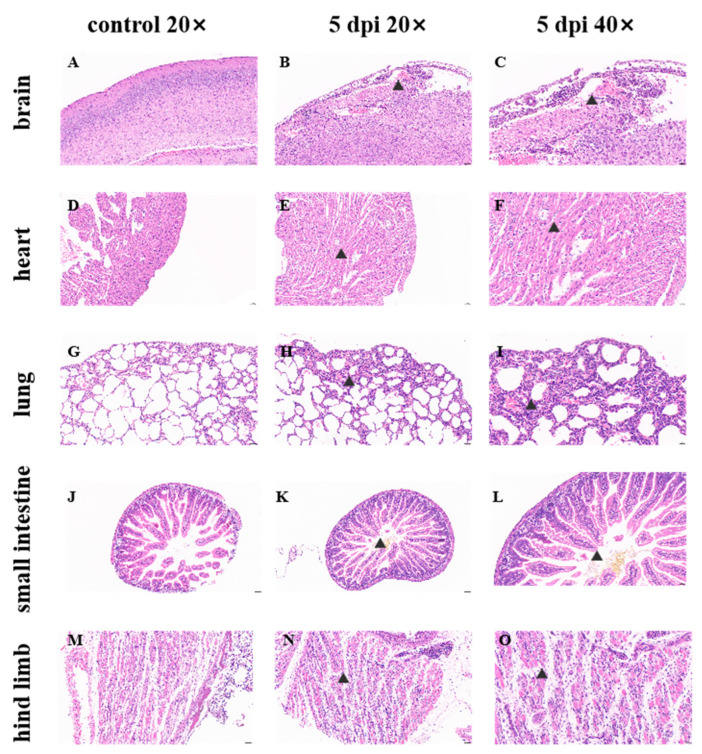
The 2-day-old IFNAR1-KO mice, infected with 10^6^ TCID_50_/mL E18 via intracranial injection, undergo histological examination and hematoxylin-eosin (HE) staining at 5 days post-infection (dpi). The first column presents the HE staining results of histopathological sections from the brain (**A**), heart (**D**), lung (**G**), small intestine (**J**), and hind limb muscle (**M**) of mice injected with a non-infected medium. The latter two sections depict the histopathological findings for the 2-day-old IFNAR1-KO mice infected with 10^6^ TCID_50_/mL E18 at 5 dpi. The second column (**B**,**E**,**H**,**K**,**N**) displays results at a magnification of 20×, while the third column (**C**,**F**,**I**,**L**,**O**) illustrates results at a magnification of 40×. These images represent findings obtained from 2 to 3 mice exhibiting similar clinical symptoms, which present representative images capturing the range of pathological features observed in our study. The section indicated by the black arrowheads in the figure shows pathological changes. To minimize bias, three independent pathologists, blinded to experimental conditions, evaluate these cases to confirm consistency.

Histopathological examination of the brain revealed no significant pathological alterations in mice injected with the culture medium (Figure 4A). In E18-infected mice, meningeal edema, disorganized connective tissue architecture, localized hemorrhage, a substantial infiltration of lymphocytes and macrophages were observed; additionally, a limited number of neurons were identified in the cortex with intensified staining and indistinct boundaries between nuclei and cytoplasm. Furthermore, a few irregular vacuoles and mild glial cell hyperplasia were noted (Figure 4B,C). The replication of E18 within brain tissue has been associated with symptoms indicative of aseptic meningitis such as fever and cerebral edema. Cardiac histopathological analysis demonstrated no significant pathological changes in the culture-injected mice (Figure 4D). Conversely, cardiac tissues from E18-infected mice exhibited minimal cytoplasmic vacuolation in cardiomyocytes along with sporadic lymphocytic infiltration surrounding interstitial vessels (Figure 4E,F). The histopathological assessment of lung tissues indicated no notable pathological changes in mice administered with culture medium (Figure 4G).

In the E18-infected mice, a modest degree of granulocyte infiltration was noted within the alveolar walls of the lungs, accompanied by slight thickening in localized areas, widening of the alveolar septa, heterogeneous alveolar sizes, and compensatory dilation of the alveolar cells. Sporadic lymphocytic infiltration was observed surrounding blood vessels, with white blood cells present within these vessels. The bronchiolar epithelium frequently exhibited flattening (Figure 4H,I). Histopathological examination of the small intestine revealed that both control and experimental groups displayed normal morphology and a regular arrangement of muscle fibers without significant inflammatory cell infiltration (Figure 4J–L). A pathological assessment of hind limb muscle tissue indicated no notable pathological changes in mice injected with medium (Figure 4M). Conversely, in E18-infected mice, hind limb muscles were severely compromised; muscle fibers varied in size and shape irregularly while exhibiting frequent breakage alongside loose and disorganized arrangements. Increased instances of muscle cell necrosis were evident along with nucleolysis and homogenous red staining. Surrounding fibroblast hyperplasia was prominent with scattered infiltrations from a limited number of lymphocytes as well as white blood cells visible within blood vessels (Figure 4N,O). The findings from pathological sections suggest that E18 infection induces severe pathology characterized by tissue lesions and inflammatory responses—particularly pronounced in the hind limb musculature—consistent with manifestations indicative of hind limb paralysis.

## 3. Discussion

Animal testing has played a pivotal role in the advancement of vaccines, antibiotics, and our fundamental comprehension of human disease mechanisms. Furthermore, animal models are essential for evaluating the efficacy and safety of a vaccine [19]. Enterovirus 18 (E18) has emerged as one of the most significant pathogenic agents linked to aseptic meningitis in children in recent years. Currently, there are no specific therapeutics or vaccines available for E18 infection, nor has a suitable animal model for studying E18 virus infection been established.

To further elucidate the pathogenic mechanisms of E18 and its replication across diverse tissues and organs, it is imperative to investigate suitable animal models. In this study, several commonly used experimental mouse strains were initially identified for preliminary testing.

Balb/c neonatal mice were employed as experimental subjects and demonstrated pronounced pathological responses during enterovirus EV71 infection, characterized by skeletal muscle injury, weight loss, and a diminished survival rate [20,21]. Preliminary findings regarding E18 infection suggest that Balb/c mice exhibit resistance to the E18 virus, demonstrating no clinical alterations even after exposure to the highest viral concentrations. Typically, animal models utilized for studying Echovirus involve immunodeficient human transgenic mice that express the human homolog of the receptor while lacking expression of the interferon-alpha/beta receptor (IFNAR). For example, a mouse model for Echovirus type 1 (EV1) was established using transgenic mice expressing human integrin very late antigen 2 (VLA-2) [22]. Furthermore, an in vivo model for Echovirus type 11 (E11) was developed using hFcRn-IFNAR mice, which are deficient in type I interferon (IFN) signaling and express the neonatal Fc receptor (hFcRn) [23].

A further comparative analysis of C57 and IFNAR1-KO mice revealed that the infection symptoms, survival rates, and weight fluctuations in the animal models subjected to both injection methods for IFNAR1-KO mice were significantly more favorable than those observed in C57 mice. This finding underscores the critical role of type I interferon in the in vivo infection process of E18 virus. The absence of the type I interferon receptor markedly heightened the incidence of pathological manifestations following infection in these mice. Moreover, the intracranial administration of IFNAR1-KO exhibited superior outcomes compared to intraperitoneal injection, indicating that the blood–brain barrier may modulate viral infection and replication.

In C57 mice, hind limb paralysis, weight loss, and even mortality were observed only when the intracranial injection of the highest concentration gradient E18 was administered; under these conditions, viral titers were detectable in the tissue homogenate. Although no positive indicators of E11 infection have been identified in any tissues of wild-type (WT) C57BL/6J mice, this finding has also been corroborated in animal models infected with E30 [17,18]. In the current study, a subset of C57 mice exhibited hind limb paralysis following the intracranial administration of E18, with E18 virus identified in various tissue sites. This finding suggests that there are variations in the susceptibility of C57 mice to different serotypes of Echovirus strains.

E18 was detected in all tissues and organs of both IFNAR1-KO and C57 mice, accompanied by corresponding pathological manifestations, indicating the extensive replication of the E18 virus in these murine models. No significant difference in viral load was observed between intracranial and intraperitoneal injections in brain tissue for both IFNAR1-KO and C57 mice, which may be attributed to the virus’s replication and transmission from the injection site to other tissues. The variation in E18 virus replication within the hearts of different mouse groups infected with E18 could be related to both the mouse strain type and injection method employed. Notably, intracranial injection yielded superior results for IFNAR1-KO mice compared to intraperitoneal injection; furthermore, when utilizing intracranial injection, IFNAR1-KO mice exhibited better outcomes than their C57 counterparts. There were no significant differences noted regarding E18 virus replication in lung tissue across various strains of mice or differing modes of injection. A comparative analysis of viral loads in the small intestine revealed that intracranial injections resulted in significantly higher viral loads for IFNAR1-KO mice relative to those receiving intraperitoneal injections. Additionally, the assessment of viral load within hind limb muscle indicated that C57 mice demonstrated a significantly greater viral load than IFNAR1-KO mice under conditions involving intraperitoneal administration.

Based on the aforementioned results, it can be concluded that variations in mouse strains and injection methodologies result in differing replication effects of E18 across various tissues. Notably, the viral load detected in numerous tissues, such as the heart and small intestine of IFNAR1-KO mice, exhibited significant discrepancies compared to other groups. While a high level of E18 virus was observed in the hind limb muscle following intraperitoneal injection in C57 mice, no individual signs of hind limb paralysis were noted; thus, the underlying reasons warrant further investigation. There was no statistically significant difference in viral load within muscle tissue between C57 mice and IFNAR1-KO mice exhibiting similar signs of hind limb paralysis. Although neurological symptoms were observed in mice, the comparable viral loads in brain tissues across groups suggest that these manifestations may involve indirect mechanisms (e.g., systemic inflammation), warranting further mechanistic investigation. Integrating findings from pathological sections across diverse tissue sites led to the conclusion that the impact of E18 virus on murine hind limb muscle is more pronounced than that on brain tissue, aligning with observations from infections caused by other Echoviruses such as E11 and E30 viruses in IFNAR-KO mice.

The clinical manifestations observed were in alignment with the viral load assessments and pathological analyses of brain tissue in the mouse model utilized in this study. Through a comprehensive evaluation of clinical signs, histopathological sections, and viral load quantifications across various tissues and organs, we established an E18 animal infection model employing 2-day-old IFNAR1-KO mice that were intracranially injected with 10^6^ TCID_50_ of E18. This study elucidates the pathological characteristics of E18 for the first time within a murine model, thereby offering valuable insights and clues for future investigations into the mechanisms underlying pathological injury as well as the protective effects conferred by antibodies.

The differential susceptibility observed between C57BL/6J and IFNAR1-KO mice, particularly the strain-specific variations in clinical signs and viral replication, suggests that intrinsic differences in viral pathogenicity may drive these phenotypes. This is consistent with established mechanisms in related Echoviruses. Pathogenicity varies significantly between isolates of the same serotype. While the prototype E9 strain (Hill) isolated from a healthy child is non-pathogenic in newborn mice, a clinical isolate (E9/Barty) obtained from the cerebrospinal fluid (CSF) of a child with aseptic meningitis exhibits high virulence in newborn Swiss albino mice [24]. Similarly, a clinical E30 isolate (A538) demonstrated lethality and susceptibility across multiple immunocompetent newborn mouse strains (ICR, BALB/c, KM, C57BL/6J) [25], whereas another E30 clinical isolate (WZ16) showed no detectable viral replication in newborn C57BL/6Jmice [18].

Specific genomic regions correlate with murine pathogenicity: comparative sequence analysis of the non-pathogenic E9/Hill strain and the virulent E9/Barty isolate identified a critical 310-aa segment including the RGD motif associated with murine virulence. Functional studies confirmed that mutating the RGD motif to RGE abolished infectivity in GMK cells. Furthermore, synthetic RGD peptides specifically inhibited the binding of the virulent E9/Barty strain but not the non-pathogenic E9/Hill strain, to GMK cells, establishing the RGD motif as a key determinant of E9 pathogenicity in this mode [26].

Collectively, these findings strongly suggest that variability in pathogenicity among isolates of the same Echovirus serotype, including the observed differences across mouse strains, may be linked to discrete genomic variations. Consequently, to elucidate the mechanistic basis for the differential pathogenicity observed with our E18 isolate in various mouse strains, a critical next step involves comparative genomic sequence analysis. Specifically, sequencing the pathogenic E18 isolate used in this study alongside non-pathogenic E18 reference or clinical strains would be essential to identify potential virulence-associated mutations, potentially within functionally critical regions analogous to the RGD motif identified in E9.

This study has several limitations. First, while we observed significant differences between experimental and control groups at the highest gradient level, the current dose–response design did not demonstrate subsequent enhancement in virus culture titers. Second, the small sample size, though sufficient for preliminary findings, warrants cautious interpretation; future validation will employ larger cohorts (*n* ≥ 5 per group) to ensure robustness. Additionally, the histopathological evaluation, while informative, lacked a standardized scoring system. Subsequent studies will adopt established criteria to improve objectivity and reproducibility. The mouse model selection also presents constraints: our study focused on three common strains for comparative analysis but did not include hFcRn-IFNAR-deficient mice, which have been shown to exhibit enhanced susceptibility to E11 infection [23]. Finally, the pathogenesis underlying the positive clinical signs in C57BL/6J and IFNAR1-KO mice remains unresolved and merits targeted investigation.

Our identification of an E18 animal infection model utilizing IFNAR1-KO neonatal mice is consistent with findings from previous studies on E11 and E30 infections. This indicates that our model can facilitate horizontal comparisons across various types of Echovirus infections. The current model primarily simulates early systemic infection, while the mechanisms underlying human meningitis require further validation. Our future research will also focus on the molecular determinants of strain-specific pathogenesis in E18. It is crucial to directly integrate the role of the host inflammatory pathway in both C57 and IFNAR1-KO mice into our experimental design. Additionally, we intend to employ this model for drug efficacy assessments as well as vaccine evaluation research related to E18.

## 4. Method

### 4.1. Ethical Statement

Animal models were established utilizing specific pathogen-free (SPF) Balb/c, C57BL/6J, and IFNAR1-KO mice. All animal experiments were approved by the Laboratory Animal Ethics Committee of Southern Medical University (Approval No. D202310-1) and were conducted in accordance with relevant guidelines and regulations.

### 4.2. Mice, Cells, and Viruses

SPF Balb/c and C57BL/6J pregnant mice were procured from the Guangdong Experimental Animal Center. The immunocompromised IFNAR1 knockout (KO) mice used in this study were generated on a C57BL/6J genetic background (Catalog Number: T005534,GemPharmatech Co., Ltd., Nanjing, China). The C57BL/6J background was matched with wild-type control group (C57 mice) to minimize confounding factors in comparative analyses [27]. This study utilizes CRISPR/Cas9 technology to edit the Ifnar1 gene, specifically targeting exons 2-6 of the IFNAR1-201 transcript (ENSMUST00000023689.10) as the knockout region, which encompasses a coding sequence of 718 base pairs. A PCR primer set was meticulously designed for validating gene expression in both wild-type and IFNAR1 knockout mice (F1: TACCCACCTGCCAAGGATTGA; R1: CAACTGAGCCATCTCTCCAGCAC; F2: CGTGGAGACTGAGCTGAGATGAAG; R2: CACTGTGTGTGTGCTGGAAATGACAG). All mice were housed in a life sciences laboratory animal facility within individually ventilated cages, lined with clean sawdust and supplemented with cotton for nesting purposes, thereby facilitating mating and subsequent pup production for further animal testing.

The E18 strain was isolated from a 2019 clinical case of E18 meningitis in an immunocompetent adult patient in Shenzhen. The virus used in experiments was at passage 3 (P3) after clinical isolation [28]. Rhabdomyosarcoma cells (RD; ATCC) were cultured in DMEM medium (Pocell), supplemented with 10% fetal bovine serum (Procell) and 1% penicillin/streptomycin, at a temperature of 37 °C in a humidified atmosphere containing 5% CO_2_. Upon achieving a cell density of 80–90%, the complete culture medium was replaced with one containing only 2% fetal bovine serum prior to viral infection, after which samples were collected following the observation of cytopathic effects (CPE). After undergoing three freeze–thaw cycles, all viruses present in the supernatant were filtered through a 0.22 μm filter and subsequently stored at −80 °C.

### 4.3. Animal Infection Experiments

Balb/c, C57BL/6J (C57), and IFNAR1-KO mice aged two days postnatal were subjected to infection with E18 using a concentration gradient of either 10^2^, 10^4^, or 10^6^ TCID_50_ (50% tissue culture infectious dose). A total volume of 50 μL of the E18 suspension was administered via either intracranial or intraperitoneal routes. Control groups received an equivalent volume of uninfected medium while being housed separately from the infected subjects.

Weight trends, survival rates, and clinical disease scores were meticulously recorded on a daily basis until day 5 post-inoculation. The clinical disease grading system was delineated as follows: 0—healthy; 1—lethargy or inactivity; 2—emaciation or weakness in the hind limbs; 3—paralysis of one hind limb; 4—paralysis of both hind limbs; 5—dying or deceased.

On the fifth day following viral infection, anesthetized mice underwent aseptic extraction of their brains, hearts, lungs, intestines, and skeletal muscles from the contralateral hind limbs. Half of the harvested tissues were homogenized and stored at −80 °C for subsequent analyses, while the remaining half was fixed in a 4% paraformaldehyde solution for tissue preservation.

### 4.4. Viral Load Measurement

Tissues from the brain, hind limb muscles, heart, lung, and small intestine were collected at 5 days post-infection (5 dpi). The samples were rinsed with sterile PBS to remove excess blood, after which 500 μL of PBS containing 1% penicillin and streptomycin was added to each tissue sample. The supernatant was then homogenized using a frozen tissue grinder and subjected to three freeze–thaw cycles. Following centrifugation, the supernatant was carefully removed for viral load determination via qPCR probe method.

Viral RNA was extracted from 50 μL of the grinding supernatant, followed by quantitative analysis via reverse transcription quantitative PCR (RT-qPCR). A standard plasmid containing a 5′ untranslated region (UTR) sequence in E18 was synthesized, along with the requisite primers and probes for qPCR as detailed below (Table 1).

### 4.5. Histopathological Analysis of E18 Infected Mice

Brain tissue, hind limb muscle, heart, lung, and small intestine from E18-infected mice were fixed in 4% paraformaldehyde for a duration of 24 h. The specimens were subsequently trimmed to achieve a standardized cross-section, conventionally processed, embedded in paraffin wax, and then sliced into sections with a thickness of 4 μm. These sections were mounted on slides treated with 3-aminopropyl triethoxysilane and stained using hematoxylin and eosin (HE). A microscopic examination was performed to elucidate the detailed histological characteristics of the tissue sections at various magnifications.

### 4.6. Statistical Analysis

Data were analyzed utilizing GraphPad Prism software (version 9.00; GraphPad, La Jolla, CA, USA) through unpaired two-tailed *t*-tests. Comparisons between the two groups were assessed using independent-sample *t*-tests. The results are presented as means ± standard deviations (SD). A *p* value of less than 0.05 was deemed to indicate a statistically significant difference between values.

## Figures and Tables

**Table 1:** Primers and probes for qPCR detection.

Primers and Probes	Sequence	Fluorophores
EV-YF	ATGGTGNGAAGAGYCTAYTGAGCT	
EV-YR	CCAAAGTAGTCGGTTCCGC	
EV-Probe	TCCGGCCCCTGAATGCGGMTAAT	5’Cy5 3’BHQ2

## Data Availability

Data is contained within the article.
